# Histological and Immunohistochemical Features of Trichoblastoma in a Sarda Breed Sheep

**DOI:** 10.3390/ani10112039

**Published:** 2020-11-04

**Authors:** Marta Polinas, Giovanni P. Burrai, Veronica Vitiello, Laura Falchi, Maria T. Zedda, Gerolamo Masala, Vincenzo Marras, Giulia Satta, Alberto Alberti, Elisabetta Antuofermo

**Affiliations:** 1 Department of Veterinary Medicine, University of Sassari, 07100 Sassari, Italy; mpolinas@uniss.it (M.P.); vevitiello@uniss.it (V.V.); lfalchi@uniss.it (L.F.); zedda@uniss.it (M.T.Z.); gemasala72@gmail.com (G.M.); alberti@uniss.it (A.A.); eantuofermo@uniss.it (E.A.); 2 Mediterranean Center for Disease Control (MCDC), University of Sassari, 07100 Sassari, Italy; 3 Department of Biomedical Sciences, University of Sassari, 07100 Sassari, Italy; marrasv@gmail.com (V.M.); giulia.satta88@gmail.com (G.S.)

**Keywords:** hair follicles, immunohistochemistry, ovine, papillomavirus, skin tumors

## Abstract

**Simple Summary:**

Skin tumors in ruminants are occasionally reported and are mostly associated with the presence of oncogenic viruses, such as papillomavirus, or to direct skin exposure to solar radiation. Spontaneous tumors with adnexal differentiation, originating from the hair follicle or its associated glands, are even more rarely reported in these species. Investigation of the histological and immunohistochemical features of a trichoblastoma (tumor arising from the hair follicle) detected in the ear of a Sarda breed sheep, allowed for the study of comparative aspects of this tumor with other domestic species in which this neoplasm is commonly reported, such as in dogs and cats, and in human beings. This work contributed to the deepening of the knowledge of ovine spontaneous tumors unrelated to the presence of papillomaviruses.

**Abstract:**

Skin tumors with adnexal differentiation are commonly reported in dogs and cats, while only anecdotal evidence is available in sheep. Here we illustrate the macroscopic, histologic, and immunohistochemical features of a cutaneous lesion with adnexal differentiation in a 6-year-old female Sarda breed sheep, surgically treated for a horn-like mass located in the left pinna. Additionally, we investigate a possible contribution of Ovine Papillomaviruses (OaPVs). Histologically, the dermis was expanded by an expansive and unencapsulated multilobulated nodule composed of cuboidal to spindle basaloid cells arranged in variably-sized cytokeratins (CK) AE1-AE3, CK 5/6 and CK 34 beta E12, p63—positive winding cords with a characteristic palisade arrangement of neoplastic cells in the periphery of the tumor. Based on these results, the cutaneous neoplasm was diagnosed as a trabecular trichoblastoma with spindle cells and rare structures resembling papillary mesenchymal bodies. Additionally, multiple enlarged sebaceous lobules clustered around dilated ducts suggestive of sebaceous gland hyperplasia were detected near the trichoblastoma. No PV DNA was found in the examined tissues, suggesting that ovine PVs are not involved in the pathogenesis of the present skin tumors with adnexal differentiation. Further investigations and efforts are required to elucidate the prevalence of skin tumors with adnexal differentiation in this species.

## 1. Introduction

Skin tumors are well-recognized entities in ruminants, both in domestic and wild species [1,2,3]. Reports of ovine cutaneous tumors are mostly referred to as squamous cell carcinoma, a condition frequently associated with poorly pigmented skin exposure to ultraviolet radiation and/or with ovine papillomaviruses (PVs) [1,4,5]. Other epithelial neoplasms—i.e., tumors of hair follicles and adnexal glands, are described as a rare event in ruminants [2,3,6]. In other species, neoplasms and tumor-like lesions arising from hair follicles are a common findings, representing 10% and 8% of skin tumors in dogs and cats, respectively, with trichoblastoma being the most common benign hair follicle tumor in dogs and the most frequent skin tumor in the rabbit [7,8,9]. In human beings, tumors of the hair follicles are considered rare and they are, as in animals, benign in most cases [10].

Adnexal tumors have a complex histological appearance, and immunohistochemistry (IHC) is a helpful tool in distinguishing follicular tumors from other cutaneous neoplasms. We herein describe the macroscopic, histologic, and immunohistochemical features of a skin tumor with adnexal differentiation in a Sarda breed sheep. Additionally, we investigate the presence of ovine papillomaviruses in the same tissues.

## 2. Materials and Methods

**Figure 1:**
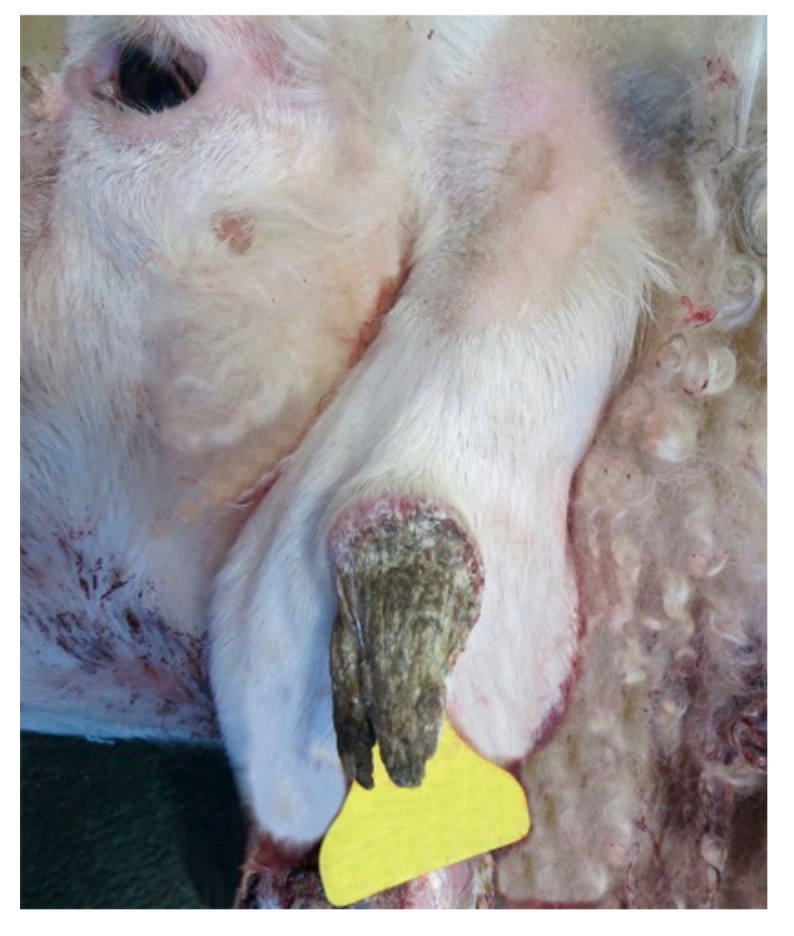
Left pinna: horn-like exophytic cutaneous mass.

A 6-year-old female Sarda breed sheep was admitted to the Veterinary Teaching Hospital of Sassari University and surgically treated for a cutaneous mass located in the left pinna. Macroscopic examination revealed a horn-like, exophytic mottled mass, measuring 4 × 1.5 × 1.2 cm, presenting an ulcer on its surface (Figure 1).

**Figure 2:**
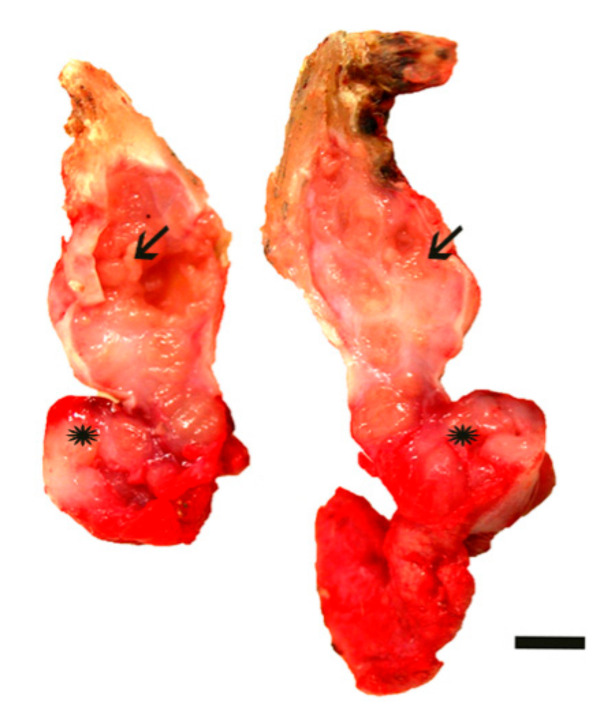
Haired skin: cut section of the cutaneous mass located in the left pinna. Two distinct multilobulated masses circumscribed by multiple white-yellowish bands and measuring 3 × 1.2 × 1 cm (arrow) and 1.3 × 1.2 × 0.5 cm (asterisk) are observed. Bar: 0.5 cm.

On the cut surface, two distinct multilobulated masses measuring 3 × 1.2 × 1 cm (nodule A) and 1.3 × 1.2 × 0.5 cm (nodule B), both circumscribed by multiple white-yellow bands, were present (Figure 2).

After sampling, the mass was divided into two separated samples; one was stored at −80 °C for molecular analysis and the other was formalin-fixed, paraffin-embedded, 3 µm-sectioned, and stained with hematoxylin and eosin (H&E) and Periodic Acid–Schiff (PAS). Additional serial sections (4 μm in thickness) were mounted on positively charged slides (Superfrost; Fisher Scientific, Rodano (MI), Italy) and used for the immunohistochemistry, following an in-house protocol previously developed [11]. Briefly, slides were immersed for 20 min in a 98 °C preheated solution (WCAP citrate pH 6; BiOptica, Milano, Italy) for antigen retrieval. After blocking the endogenous peroxidase and non-specific binding (Peroxidase-Blocking Solution—Dako REAL—and 2.5% normal horse serum—Vector Laboratories), tissues were incubated overnight at 4 °C with the primary antibodies listed in Table 1.

Sections were then incubated with an anti-mouse/rabbit secondary antibody (ImmPRESS reagent kit—peroxidase—MP-7500; Vector Laboratories, Burlingame, CA, USA) for 30 min at room temperature and treated with chromogen 3,3′-Diaminobenzidine (DAB) (ImmPACT DAB; Vector Laboratories). Tissues were then counterstained with hematoxylin, dehydrated, and mounted (Eukitt Mounting Medium; BiOptica, Milano, Italy). Appropriate positive and negative controls were included for each antibody. Slides were examined under a light microscope (Nikon Eclipse 80i) and photographed.

In order to evaluate the presence of PV DNA, total DNA was extracted from 25–30 mg tissue samples (DNeasy Blood & Tissue isolation kit; Qiagen, Milano, Italy), according to the manufacturer’s instructions. Samples were subjected to rolling circle amplification (RCA) (TempliPhi 100 Amplification Kit; GE Healthcare, Milano, Italy), as previously described [4]. Furthermore, samples were tested by PCR with primers specific to the PV types infecting sarda breed sheep (OaPV3 and OaPV4) [4,12]. Additionally, samples were tested with the universal primers pair FAP59/64, targeting a conserved region of the papillomaviral L1.

## 3. Results

**Figure 3:**
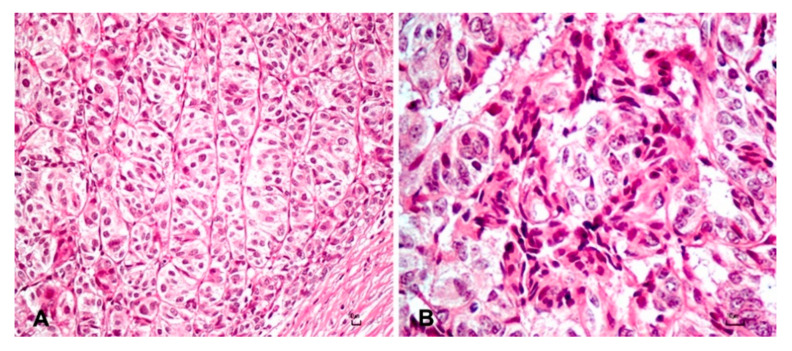
(**A**) Columns and trabeculae of basaloid epithelial cells of the trichoblastoma. Hematoxylin and eosin (H&E). Bar 10 µm. (**B**) Foci of mesenchymal cells closely associated with neoplastic epithelial cells resembling papillary mesenchymal bodies. H&E. Bar 10 µm.

Histological examination of the nodule A revealed an exophytic, well-demarcated, multilobulated, and unencapsulated mass that expanded the dermis and partially effaced the adnexa. The neoplasm was densely cellular and composed of cuboidal to spindle basaloid cells arranged in variably sized winding columns and trabeculae sustained by a finely hyalinized fibro-vascular stroma (Figure 3A).

Occasionally, foci of mesenchymal cells were scattered in the stroma closely associated with neoplastic epithelial cells and resembling follicular papillae (Figure 3B). The neoplastic basaloid cells showed distinct cell borders, in cuboidal to fusiform shape, and scant to moderate pale eosinophilic cytoplasm (Figure 3A) with a slightly pleomorphic, oval nucleus with finely stippled chromatin and a small single nucleolus. Atypical features, such as anisocytosis and anisokaryosis, were mild. Mitotic figures were 12 in 10 consecutive high-power field (HPF) (field area of 2.37 mm^2^) and sometimes atypical. Moreover, the intact epidermis often formed rete ridges, and the scattered and multifocal—mostly within the stratum spinous—presence of koilocytes with moderately swollen with a large perinuclear clear cytoplasm halo and pyknotic nuclei was observed. Koilocytes were defined according to Goldschmidt and colleagues (2018) [13]. Cells of the stratum granulosum contained numerous large, irregularly rounded keratohyalin granules while, diffusely, the stratum corneum is expanded by several layers of laminar keratin (orthokeratotic hyperkeratosis).

**Figure 4:**
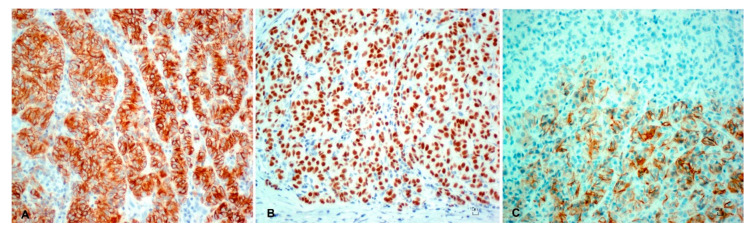
Immunohistochemistry. DAB and hematoxylin (**A**) Cytokeratin 5/6 strong and diffuse cytoplasm immunostaining of epithelial basaloid trichoblastic cells. DAB and hematoxylin. Bar 10 µm; (**B**) P63 strong and diffuse nuclear expression of epithelial basaloid trichoblastic cells. Bar 10 µm; (**C**) Moderate and focal cytoplasmic immunoreactivity of CD56 was multifocally detected in 8–10% of epithelial basaloid trichoblastic cells. Bar 10 µm.

Neoplastic cells showed a diffuse and strong cytoplasmic immunoreactivity for cytokeratin (CK) AE1-AE3, CK 5/6, and CK 34 beta E12 (Figure 4A). Nuclear expression of p63 was strong and diffuse in 100% of neoplastic cells (Figure 4B), and the mean Ki-67 labeling index was 24.3%. A moderate cytoplasmic immunoreactivity expression of CD56 was multifocally detected in 8–10% of neoplastic cells (Figure 4C). Melan-A was negative.

Based on morphological features observed at histopathology and immunohistochemical properties of neoplastic cells, a diagnosis of trichoblastoma was made. Results of immunohistochemical examination are summarized in Table 2.

**Figure 5:**
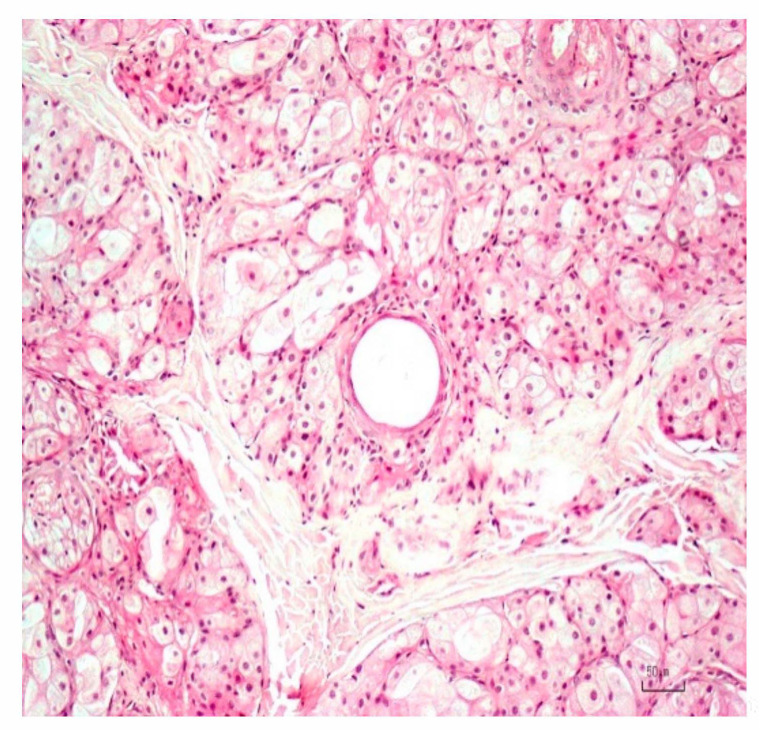
Enlarged sebaceous lobules oriented around a small dilated duct lined by squamous keratinizing epithelium characterized the sebaceous gland hyperplasia. H&E. Bar 50 µm.

The adjacent nodule B was characterized by multiple enlarged sebaceous lobules, oriented around dilated ducts lined by squamous keratinizing epithelium (Figure 5).

The lobules were composed of polygonal sebaceous cells with abundant cytoplasmic containing lipidized vacuoles and a central nucleus. A single layer of basaloid reserve cells, with scant eosinophilic cytoplasm and a central nucleus, was located peripherical to lobules. Due to the described characteristics, the diagnosis was consistent with sebaceous gland hyperplasia.

## 4. Discussion

Trichoblastoma is classified in the following histological subtypes based on displayed morphological features: ribbon type, medusoid, trabecular, spindle, granular, solid/cystic, and with outer root sheet differentiation, sometimes presenting overlapping features belonging to two or more subtypes [13,14,15,16]. The latter situation likely represents the present case, where a prominent trabecular pattern was intermingled with spindle cells and rare structures resembling papillary mesenchymal bodies described in dogs [15,16]. Trichoblastoma arises indeed from the primitive hair germ and associated dermal papilla, which are recognizable in dogs as basaloid cells and follicular papillary mesenchymal bodies, respectively [15,16]. In the case we are presenting, rare papillary mesenchymal bodies representing abortive follicular papillae and evocative of the relationship between primitive hair germ and the papilla, were presumably identified by their morphological characteristics and by lack of positivity for p63, consistent with that reported by other authors in dogs [16]. Trichoblastoma is an epithelial neoplasm deriving from basal cells, that expresses markers for epithelial and basal origin such as CK AE1/AE3, CK5/6, CK 34 beta E12, and p63, both in human and in veterinary medicine [17,18]. Trichoblastoma neoplastic cells of the present case stained positively for cytokeratins and p63, showing staining features similar to what reported in the literature (Figure 4A,B).

Differential diagnoses for trichoblastomas are limited due to the characteristics of morphological features that allow for a straightforward diagnosis, and these include basal cell carcinoma (BCC) and apocrine solid-cystic ductular adenomas, particularly for the spindle cells type trichoblastoma of dogs and cats, and inferior type tricholemmoma [15]. Immunohistochemistry has proven to be a useful tool to distinguish between BCCs and trichoblastomas in humans. A recent paper confirmed the role of Ki67 in differentiating the two neoplasms, by the demonstration of lower expression of Ki67 in trichoblastomas cells (about 20%) compared to BBC cells (about 100%) [19]. A similar low percentage of Ki67 expression combined with mild to moderate anisokaryosis and anisocytosis, the absence of haphazard polarity of neoplastic cells, and lack of necrotic foci of the present case ruled out a malignant basal cell neoplasm. Solid-cystic ductular adenomas, together with basal cell carcinomas and trichoblastomas, have been included for many years in the basal cell tumor group, due to their overlapping histological features [15]. A clue in differentiation between trichoblastoma and apocrine ductular adenoma in the present case was the absence of morphologic features typically reported in solid-cystic ductular adenomas, rather than immunohistochemical properties of the neoplasm [15]. In fact, the absence of ductular structures, cystic degeneration and necrosis, and lack of contiguity with epidermis at histology allowed us to discard an apocrine origin of the neoplasm. Furthermore, the inferior type tricholemmoma was excluded by PAS negative staining of all neoplastic cells, including the peripheral cells arranged in palisade [15].

The neuroendocrine differentiation marker CD56 is frequently part of the IHC panel for the identification of Merkel cell tumor (MCC) in human pathology. This cutaneous neoplasm can display similar features to tumors of basal cell origin. In a recent work on the differential expression of CD56 and CK5/6 in basal cell carcinomas (BCCs) and MCCs in humans, a focal and moderate immunoreactivity for CD56 and a strong and diffuse positive labeling for CK5/6 was detected in BCCs, whereas the opposite was observed in MCCs [20]. The latter pattern was also reported as a faint immunoreactivity for cytokeratin in MCC cells of dog and cat [21,22]. Consistently with these examples, the scattered expression of CD56 in trichoblastoma neoplastic cells was interpreted as a feature that could be displayed by a tumor of basal cell origin. Although still debated, papillomaviruses are variably involved in non-melanoma skin cancer [23]. Moreover, in Sarda breed sheep, two PV types, namely Ovis aries Papillomavirus 3 (OaPV3), and 4 (OaPV4), were fully sequenced and enrolled in the aetiology of ovine squamous cell carcinoma and fibropapillomas [4,5,12]. In our study, we ruled out the presence of PV DNA within epithelial cells, confirming that the presence of enlarged keratohyaline granules and koilocytes are suggestive but not sufficient to predict PVs involvement.

## 5. Conclusions

Tumors with adnexal differentiation are only occasionally reported in small ruminants. Differently from most of the ovine squamous carcinomas and fibropapillomas related to the presence of oncogenic viruses, the etiology of the present case is not associated with ovine papillomaviruses. Moreover, comparative aspects with other domestic species and with humans allowed us to detect common histological features of these rarely reported neoplasms in ruminants, adding new information to the knowledge of spontaneous tumors in this species and expanding, at the same time, the panel of antibodies suitable for immunohistochemical characterization of uncommon lesions. However, future work is needed to acquire a comprehensive view of viral and non-viral cutaneous proliferative lesions in Sarda breed sheep.

## Figures and Tables

**Table 1:** Panel of primary antibodies and their dilutions used for immunohistochemistry.

Antigen	Dilution	Clone and Source
CK AE1/AE3 ^1^	1:200	Mouse Monoclonal Anti-Cytokeratin Clone AE1/AE3, Dako
CK 34 beta E12 ^1^	1:200	Mouse Monoclonal Anti-Cytokeratin (clone 34ßE12), Ventana
CK 5/6 ^1^	1:200	Mouse Monoclonal Anti-Cytokeratin 5/6 (D5/16B4), Ventana
p63	1:100	Mouse Monoclonal anti-p63 (4A4), Ventana
Ki67	1:200	Mouse Monoclonal Anti-Human Ki-67 Clone MIB-1, Dako
CD56 ^2^	1:100	Rabbit Monoclonal Antibody (clone MRQ-42), Cell Marque
Melan-A	1:100	Mouse Monoclonal Anti-Human Melan-A (Clone A103), Dako

^1^ CK, cytokeratin; ^2^ CD, cluster of differentiation.

**Table 2:** Immunolabeling patter of the examined trichoblastoma.

Tumor	CK AE1/AE3	CK 34BE12	CK 5/6	p63	KI-67	CD56	Melan-A
Trichoblastoma	++	++	++	++	+	+	−

++ = strong and diffuse signal; + = moderate signal in < 30% of cells; − = absence of signal.
